# The influencing factors of clinical nurses’ problem solving dilemma: a qualitative study

**DOI:** 10.1080/17482631.2022.2122138

**Published:** 2022-09-18

**Authors:** Yu Mei Li, Yi Fan Luo

**Affiliations:** a Department of Nursing, Shanghai Pulmonary Hospital, Tongji University, Shanghai, China; b Tongji University School of Medicine, Shanghai, China; c Beijing Tiantan Hospital, Capital Medical University, Beijing, China

**Keywords:** Nurse, problem solving, qualitative research

## Abstract

**Purpose:**

Problem solving has been defined as “a goal-directed sequence of cognitive and affective operations as well as behavioural responses to adapting to internal or external demands or challenges. Studies have shown that some nurses lack rational thinking and decision-making ability to identify patients’ health problems and make clinical judgements, and have poor cognition and response to some clinical problems, easy to fall into problem-solving dilemma. This study aimed to understand the influencing factors of clinical nurses’ problem solving dilemma, to provide a basis for developing training strategies and improving the ability of clinical nurses in problem solving.

**Methods:**

A qualitative research was conducted using in-depth interviews from August 2020 to December 2020. A total of 14 participants from a tertiary hospital in Shanghai, China were recruited through purposive sampling combined with a maximum variation strategy. Data were analysed with the conventional content analysis method.

**Results:**

Three themes and seven subthemes were extracted: nurse’s own factors (differences in knowledge structure and thinking, differences in professional values, poor strain capacity); improper nursing management (low sense of organizational support, contradiction between large workload and insufficient manpower allocation); patient factors (the concept of emphasizing medicine and neglecting to nurse, individual differences of patients).

**Conclusion:**

The influencing factors of clinical nurses’ problem-solving dilemma are diverse. Hospital managers and nursing educators should pay attention to the problem-solving of clinical nurses, carry out a series of training and counselling of nurses by using the method of situational simulation, optimize the nursing management mode, learn to use new media technology to improve the credibility of nurses to provide guarantee for effective problem-solving of clinical nurses.

## Introduction

Nursing education in China can be divided into two main levels: vocational education and higher education. Vocational education includes technical secondary schools and junior colleges, while higher education includes undergraduate, master’s and doctoral education. Vocational education aims at training students to master basic nursing service skills and to be able to take the post to engage in daily nursing work (Sun & Zong, 2017). Higher nursing education started late, and undergraduate education has always followed the “three-stage” education model of clinical medicine (basic medical courses, specialized courses and clinical practice). Most courses are centred on subject knowledge, and all clinical practice takes the form of centralized practice (Li, 2012). The training goal of nursing postgraduates is gradually expanding from academic master to professional master. The curriculum mainly includes classroom teaching and clinical practice. The classroom teaching contents include public courses (political theory, foreign languages, etc.), professional basic courses (advanced health assessment, pharmacotherapy, pathophysiology, evidence-based nursing, medical statistics or clinical epidemiology), specialized courses (advanced nursing practice theory) and Academic activities . The goal of nursing doctoral training is to cultivate high-level nursing research talents, focusing on the cultivation of scientific research ability rather than clinical practice ability. The curriculum includes ideology and politics, basic theory, research methods, specialized courses, development frontier, scientific writing, etc (Luo et al., 2018). There are some problems in the training mode and curriculum, such as theory and practice are out of touch, traditional lecture-based classroom teaching makes students passively accept knowledge, students attach importance to theory over practice, knowledge input to ability output, professional study to humanities knowledge. Nursing students receive no theoretical and/or practical training in problem solving before entering the clinical setting, so there is not a starting point for these nurses to clinical dilemmas in their professional life.

With the development of medicine, people pay more attention to health and have higher requirements for nursing service ability (Yang, Ning, et al., 2018). The National Nursing Development Plan (National Development and Reform Commission, 2017) points out that it is necessary to strengthen the construction of nurse teams, establish nurse training mechanisms and improve the professional quality and service ability of nurses. However, in the face of increasingly complex and changeable clinical environment, nurses are still lacking in problem-solving thinking and ability, and often fall into the dilemma of problem solving (Li et al., 2020).

Typical decision theory approaches to the identification of problem solving in nursing have viewed the process as a series of decision formulations that include: decisions about what observations should be made in the patient situation; decisions about deriving meaning from the data observed (clinical inferences); and decisions regarding the selection of action to be taken that will be of optimal benefit to the patient (McGuire, 1985). Information processing theory describes problem solving as an interaction between the information processing system (the problem-solver) and a task environment, which can be analysed as two simultaneously occurring sub-processes of “understanding” and “search” (VanLehn, 1989). Individuals collect the stimulus that poses the problem in the understanding process, forming the internal representation of the problem, transforming the problem stimulus into the initial information needed in the search process, and then producing mental information structures for the understanding of the problem, which making individuals distinguish the nature of the problem and clarify the goal of the problem. The mental information structures drive the search process that enables the individual to find or calculate the solution to the problem. This process starts with the nurse identifying the clinical problem and continues until the decision is made to resolve the problem (Taylor, 2000). Clinical problem solving requires nurses to have a variety of cognitive strategies, which involves nurses’ knowledge, experience, and memory process. Nurses must recognize the current problem and use all available knowledge and experience to transform the problem into their internal problem representation, and then set goals and search for strategies that can achieve the goal (Mayer & Wittrock, 1992). In today’s complex clinical environment, nurses need to be able to solve problems accurately, thoroughly, and quickly. Nurses who can solve problems efficiently have fewer medical errors (Babaei et al., 2018), and the level of nursing skills and empathy are higher (Ay et al., 2020; Bayindir Çevik & Olgun, 2015). To cultivate nurses’ problem solving thinking and ability, it is necessary to better understand the influencing factors of problem solving dilemma. However, these cannot be obtained by observing nurses’ behaviour in their work, and cannot be obtained through quantitative research either. Exploring the thinking process involved in nurses’ work through qualitative interviews is an effective way to understand the influencing factors of nurses’ problem solving. Given this, this study used qualitative research methods to deeply analyse the influencing factors of clinical front-line nurses’ problem solving dilemma, to provide a basis for making relevant strategies to cultivate nurses’ thinking and ability of problem solving.

## Methods

### Study design

A qualitative study based on in-depth interviews was conducted to obtain influencing factors of nurses’ problem-solving dilemma.

### Settings and participants

Purposive sampling combined with a maximum variation strategy was used to identify and select information-rich participants related to the research phenomenon. Maximum variation was achieved in terms of participants’ gender, education level, professional title, marital status, seniority, and administrative office, respectively. The study was conducted between August 2020 to December 2020 in a tertiary hospital in Shanghai, China. The inclusion criteria were a nurse practicing certificate of the People’s Republic of China and within the valid registration period; having been engaged in clinical nursing work for at least 1 year and still engaged in clinical nursing work; clear language expression, able to clearly describe the solution and feelings of clinical problem solving; informed consent to this study and voluntary participation. The exclusion criterion were on leave during the study period (personal leave, maternity leave, sick leave, etc.); out for further study or came to the hospital for further study; confirmed or suspected mental illness and psychotropic medicine users. Purposive sampling continued until thematic saturation was reached during data analysis.

### Data collection

Face-to-face, a semi-structured interview was used to collect information. All interviews were conducted in the lounge to ensure quiet and undisturbed by a female postgraduate nursing student with the guidance of her master tutor. Initially, an interview guide was developed based on literature review and expert consultation including about five predetermined questions: What thorny problems have you encountered in clinical work or have a great impact on you? How did you solve it? Why take such a solution? What is the biggest difficulty encountered in the process of problem solving? How does it affect you? How do you feel in the process of problem solving? Before the interview, the consent of the interviewee was obtained and then the researcher fully explains to the interviewees and starts with a friendly chat to allay the interviewees’ worries. During the interview, the researcher listened carefully and responded in time, always maintaining a neutral attitude, without any inducement or hint, if necessary, giving encouragement and praise to support the expression of the interviewees, and to record the interviewees’ facial expressions, physical movements and emotional responses in time. At the same time, a recording pen was used to ensure that the interview content was recorded accurately and without omission. The interview time for each person was 30 to 40 minutes.

### Data analysis

After each interview, the researcher wrote an interview diary in time to reflect on the interview process and transcribed the interview content into words within 24 hours, then the researcher made a return visit by phone the next day to confirm that the information is correct. The seven-step method of Colaizzi’s phenomenological analysis method (Table I) was adopted to analyse the collected data(Colaizzi, 1978). Two researchers collated the original data, independently coded, summarized this information as themes, and organized a research group meeting once a week to discuss and reach a consensus.

**Table I.** 7 steps of Colaizzi’s phenomenological analysis method.

Step	Description
1.Familiarization	The researcher familiarizes him or herself with the data, by reading through all the participant accounts several times.
2.Identifying significant statements	The researcher identifies all statements in the accounts that are of direct relevance to the phenomenon under investigation.
3.Formulating meanings	The researcher identifies meanings relevant to the phenomenon that arise from a careful consideration of the significant statements. The researcher must reflexively “bracket” his or her pre-suppositions to stick closely to the phenomenon as experienced (though Colaizzi recognizes that complete bracketing is never possible).
4.Clustering themes	The researcher clusters the identified meanings into themes that are common across all accounts. Again bracketing of pre-suppositions is crucial, especially to avoid any potential influence of existing theory.
5.Developing an exhaustive description	The researcher writes a full and inclusive description of the phenomenon, incorporating all the themes produced at step 4.
6.Producing the fundamental structure	The researcher condenses the exhaustive description down to a short, dense statement that captures just those aspects deemed to be essential to the structure of the phenomenon.
7.Seeking verification of the fundamental structure	The researcher returns the fundamental structure statement to all participants (or sometimes a subsample in larger studies) to ask whether it captures their experience. He or she may go back and modify earlier steps in the analysis in the light of this feedback.

### Ethical considerations

This study was approved by the Ethics Committee of the Shanghai Pulmonary Hospital, Affiliated to Tongji University, project number: K16-252. Before the interview, the researcher explained the purpose and significance of the study to each interviewee in detail and obtained the informed consent of them on a voluntary basis and all of the interviewees signed informed consent forms. To protect the privacy of each interviewee, their names are replaced by numbers (e.g., N1, N2), and the original materials and transcribed text materials involved are kept by the first author himself, and all materials are destroyed after the completion of the study.

## Results

There was no new point of view when the 13th nurse was interviewed, and there was still no new point of view when one more nurse was interviewed, the interview was over, 14 nurses were interviewed. Three themes and seven subthemes were extracted. The characteristics of the participants (*N* = 14) are provided in Table II.

**Table II.** Participant characteristics (N = 14).

Characteristics		*n* (%) or M ± SD; range
Age (years)		30.29 ± 8.49;22 ~ 48
Working years		9.71 ± 9.25; 1 ~ 29
Gender		
	Male	1(7.14%)
	Female	13 (92.86%)
Educational level		
	Junior college student	4 (28.57)
	Undergraduate student	10 (71.43%)
Professional title		
	Junior nurse	8 (57.14%)
	Nurse Practitioner	1 (7.14%)
	Nurse-in-charge	4 (28.57%)
	Associate Professor of nursing	1 (7.14%)
Marital status		
	Married	6 (42.86%)
	Unmarried	8 (57.14%)
Department		
	Department of infectious diseases	3 (21.43%)
	Medical department	6 (42.86%)
	Intensive care unit	3(21.43%)
	Surgical department	2 ()14.29%

## Nurses’ own factors

### Differences in knowledge structure and thinking

Differences in the structure of prior knowledge and way of thinking will affect nurses’ processing of clinical data, thus affecting their clinical decision-making. The nurses made a wrong judgement of the condition because of the solidified thinking that postoperative nausea and vomiting symptoms were side effects of narcotic drugs and the lack of overall control and understanding of the patient’s condition.

There was a patient who came back after surgery with nausea and vomiting, the first thing that went through my mind, is the drug side effects, so I didn’t pay much attention, as is often the case, the most common cause of postoperative nausea and vomiting is anesthetic drug side effects, but later found to be cerebral infarction, this kind of situation I find it hard to recognize.

### Differences in professional values

Professional values of nurses are accepted codes of conduct internalized by nursing professionals through training and learning (Pan, 2016). Negative professional values are easy to lead to problem solving dilemma. Some nurses think nursing is just a service.

The work is difficult to do, everything is the nurse’s fault, the nurse must apologize and put up with the patient’s scolding, nursing is a service industry, sometimes I am really wronged.” There are also nurses who believe that nursing work can reflect their personal value, and solving problems successfully will bring them a sense of achievement.

Although the nursing work is very intense, I live a full life every day. I feel a sense of accomplishment and pride that I can solve the problems of patients and discharge them smoothly through my work.

### Poor strain capacity

Nursing work is patient-centred holistic nursing, the current clinical situation is complex and changeable, requiring nurses must have good strain capacity, and can “be anxious about what the patient needs, think what the patient thinks, and solve the patient’s difficulties.”

All patients are self-centered, and they don’t care whether you (the nurse) are busy or not. For example, once I gave oral medicine to a patient, a patient in the same ward was in a hurry and asked me to help him call his son. I was busy handing out the medicine and did not help. As a result, the patient was very dissatisfied and complained to the head nurse.

The 20-bed patient went through the discharge formalities but was still lying in the hospital bed. when the new patient arrived and she didn’t leave, I went to urge her to leave the hospital, she suddenly got angry and scolded me, I don’t know what to do.

### Improper nursing management

#### Low sense of organizational support

Organizational support is an important resource for clinical nurses in the process of problem solving (Poghosyan et al., 2020). Low sense of organizational support will hinder nurses’ problem solving.

The style of leadership and the atmosphere of the department are very important. in a department I rotated before, the leader was too strict to listen to your explanation, and the atmosphere of the department was not good. I couldn’t find help when I encountered problems. When I have a conflict with a patient, the leader will only criticize me, which makes me feel helpless.

Sometimes there will be a conflict with patients due to the bed turnover problem, and the patient will not listen to your explanation and turn around to complain, the nurse will be responsible for such things. In severe cases, even violent incidents will be encountered and the personal safety can not be guaranteed.

#### Insufficient allocation of manpower

Although the total number of nurses has increased substantially, there is still a shortage of human resources under the rapidly increasing workload (Guo et al., 2021).

When I was on the night shift and I encountered the critical moment of rescuing patients, I had to call an anesthesiologist, a doctor on duty, a nurse on duty simultaneously, an observation of the patient’s condition to prevent accidents was needed, I also have to race against time to give the patient ECG monitoring and oxygen inhalation. When the doctor came, he also criticized me that the first-aid equipment was not in place (crying).

According to the normal nurse-patient ratio, each nurse takes care of eight patients, and now there are not only eight patients, but also with extra beds and a fast turnover, and sometimes a nurse is responsible for more than 12 patients

### Patient factors

#### The concept of emphasizing medicine and neglecting to nurse

There is a deviation in society’s cognition of the profession of nurses, which believes that nurses are the “legs” of doctors, and nurses’ work is to help doctors run errands, give injections and give fluids. This concept not only leads to nurses’ lack of due respect, but also hinders nurses’ professional identity, and has a great negative impact on nurses’ problem-solving (Gao et al., 2015).

The patient did not dare to tell the doctor something he was not satisfied with, but complained directly to the nurse. For example, if the patient did not want to do some tests, he would scold the nurse. The nurse explained to him that he would not listen. But when the doctor came, he smiled and refused to admit that he cursed nurses, and he would frame the nurse. 90% of the patients would be willing to listen to the doctor.

Sometimes the patient says he was not feeling well, and I know the patient’s condition. I will give her some reasonable explanations, but the patient does not accept it. She is satisfied only when the doctor come to see her. In the final analysis, the patient just don’t believe us. No matter how much I explain to her, it is not as effective as the doctor’s glance at her.

#### Individual differences of patients

There are differences in patients’ personality characteristics, cultural background, views on nurses and state of an illness, these individual differences are also the reasons for nurses’ problem-solving dilemma (Chan et al., 2018).

Some cancer patients are in a period of anger, and it is very difficult to communicate with him. When I see him angry and lose his temper, I will not talk to him and just leave.”

Patients have different cultural levels and different social backgrounds. Sometimes I can’t talk too deeply. If patients are a little more educated, it will be easier for us to communicate with them, and some patients can’t understand anything we say.”

## Discussion

### Multiple factors affecting clinical nurses’ problem-solving dilemma

The reasons for nurses’ failure in problem solving are mainly in the process of understanding the problem, the search process driven by the psychological information structure, and the problem or loss of balance in the process of implementing the plan. In the process, the three factors of nurses, management and patients all played an important role. Nurses’ knowledge structure and thinking loopholes led to the deviation of nurses’ internal representation of the problem (Jonassen, 2005). Poor professional values and low sense of organizational support can lead to nurses’ negative orientation and attitude towards problems (Poghosyan et al., 2020; X. Wang et al., 2018). The manpower allocation of nurses, patients’ emphasis on medical treatment over nursing care, and individual differences mainly increase the complexity and difficulty of nurses’ problem-solving task environment as external factors. The three factors work together on the problem-solving of clinical nurses, which leads to the dilemma of problem-solving.

### Implementing situational simulation training to improve the comprehensive quality of nurses

At present, the overall quality and ability of nurses cannot meet the requirements of systematic, effective and rapid problem-solving. It is necessary to strengthen the construction of nurses to improve nurses’ problem-solving ability. Some studies have shown that situational simulation class can improve students’ knowledge, experience, psychological quality and other abilities (Mohammad, 2020). It is suggested that nursing educators should explore targeted situational simulation teaching and strengthen the relationship between classroom teaching and clinical practice through situational simulation, and to build a novel, perfect and clinical knowledge network for nurses. Secondly, emergency situational simulation teaching should be carried out to enable nurses to experience emergency situations from different angles, so as to improve their thinking, skills and timeliness in dealing with emergencies (Zhang et al., 2019). The content of professional values training should also be added to the situational simulation class in order to cultivate nurses’ positive, accessible and stable professional values and promote their positive orientation and attitude when facing problems (Skeriene, 2019).

### Optimize nursing management and improve nurses’ working experience

Through interviews, it is found that nursing management factors have caused nurses’ problem-solving dilemma to a certain extent, which needs to be optimized according to the specific problems existing in nursing management to help nurses deal with the problems and solve the dilemma effectively. The total number of registered nurses in China exceeded 4.7 million in 2021, an increase of 1.46 million from 3.24 million in 2015, an increase of 45% (Deng et al., 2019]. However, there is still a large workload and underallocation of manpower, which may be due to the unreasonable distribution of human resources between time periods and departments. Hospitals and nursing managers can use the hospital information system to evaluate the nursing workload, and allocate nursing human resources reasonably according to the evaluation results (H. Yang et al., 2019), so as to avoid nurses falling into the dilemma of problem solving due to long-term overloaded work. In addition, it is necessary to create a harmonious departmental atmosphere for nurses, create a supportive departmental environment (Aghaei et al., 2020), and strictly ensure the safety of nurses’ practice and put an end to the occurrence of violence. Timely and strong organizational support can reduce the painful feelings of nurses caused by adverse events (Stone, 2020). and help them to solve problems actively.

### Using new media to improve the image and credibility of nurses

There is a bias in social cognition of the profession of nurses, and some negative media reports mislead patients, resulting in social stereotypes of nurses (L. Q. Wang et al., 2021). It is necessary to make full use of new media to objectively introduce the nursing profession to the public, publicize outstanding nursing figures and typical deeds, make the public realize the important role of nurses in health care, and create an atmosphere of understanding and supporting nurses in the whole society to enhance the image and credibility of nurses and help nurses deal with problems and solve difficulties effectively (Falkenstrom, 2017).

## Limitations and strengths of the study

The limitation is that the transferability of this study’s results may be limited as a result of including a small number of participants and the participants all worked in the same hospital in Shanghai. More participants in different cities and hospitals could have increased the variety of the descriptions and experiences. The strength is that the use of purposive sampling facilitated inclusion of participants from a range of demographic groups. The use of maximum variation strategy facilitated that the participants covered different gender, education level, professional title, marital status, seniority and department, which helped to increase the representativeness of sample.

## Implications for practice

This study provides an in-depth exploration of the problem solving dilemmas of clinical nurses in China and provides valuable insights into the continuing education of nurses. These insights shine a light on areas that warrant further investigation and need to be improved in continuing education of nurses. It is of great significance to improve nurses’ problem-solving ability, improve nurses’ professional quality, effectively solve patients’ medical treatment and health problems, and improve patients’ experience of seeking medical treatment.

## Conclusion

Through the semi-structured interview, it is found that the problem-solving dilemma of clinical nurses is affected by many factors. Nurses themselves should be confident, self-improvement, constantly learning and enterprising to improve their own ability, and be good at using new media to improve nurses’ image and credibility. Hospitals, nursing administrators and nursing educators should take corresponding measures to improve the knowledge structure of nurses, cultivate nurses’ positive professional values and adaptability, and give full organizational support to nurses. optimize the allocation of nursing human resources to provide a strong guarantee for nurses to deal with problems solving dilemma.

## References

[cit0001] Aghaei, H., Sadat Asadi, Z., Mirzaei Aliabadi, M., & Ahmadinia, H. (2020). The relationships among occupational safety climate, patient safety climate, and safety performance based on structural equation modeling. *Journal of Preventive Medicine and Public Health = Yebang Uihakhoe Chi*, 53(6), 447–7. 10.3961/jpmph.20.350 33296585 PMC7733748

[cit0002] Ay, F., Polat, Ş., & Kashimi, T. (2020). Relationship between the problem-solving skills and empathy skills of operating room nurses. *The Journal of Nursing Research: JNR*, 28(2), e75. 10.1097/jnr.0000000000000357 31856024

[cit0003] Babaei, M., Mohammadian, M., Abdollahi, M., & Hatami, A. (2018). Relationship between big five personality factors, problem solving and medical errors. *Heliyon*, 4(9), e00789. 10.1016/j.heliyon.2018.e00789 30238063 PMC6143678

[cit0004] Bayindir Çevik, A., & Olgun, N. (2015). Do problem-solving skills affect success in nursing process applications? An application among Turkish nursing students. *International Journal of Nursing Knowledge*, 26(2), 90–95. 10.1111/2047-3095.12043 25098504

[cit0005] Chan, E. A., Wong, F., Cheung, M. Y., & Lam, W. (2018). Patients’ perceptions of their experiences with nurse-patient communication in oncology settings: A focused ethnographic study. *PloS One*, 13(6), e0199183. 10.1371/journal.pone.0199183 29912967 PMC6005521

[cit0006] Colaizzi, P. F. (1978). Psychological research as a phenomenologist views it. In R. S. Valle & M. King (Eds.), *Existential-phenomenological alternatives for psychology* (pp. 48–71). Oxford University Press.

[cit0007] Deng, J., Ye, X. C., & Liang, L. L. (2019). Status quo of mental workload of nurses in some hospitals in Shanghai and its influencing factors. *Chinese Nursing Research*, 33(3), 399–403. 10.1097/ANS.0000000000000156

[cit0008] Falkenstrom, M. K. (2017). A qualitative study of difficult nurse-patient encounters in home health care. *ANS. Advances in Nursing Science*, 40(2), 168–183. 10.1097/ANS.0000000000000156 27798435

[cit0009] Gao, D. D., Li, J., Xu, C. J., Lu, Y. H., Yang, X., Gao, F. L., Wang, J. Y., & Han, J. H. (2015). Study on the nurses’ cognition on sustainable development of high quality nursing care in Beijing. *Chinese Hospital Management*, 35(12), 77–79 https://kns.cnki.net/kcms/detail/detail.aspx?dbcode=CJFD&dbname=CJFDLAST2016&filename=YYGL201512035&uniplatform=NZKPT&v=tzIOR3BK5XPwrEGRv-zg3d5epVrkEKaR_t8heS4dRsIsdbx4lqFmp6xZBjJK0b-m.

[cit0010] Guo, J., Li, C. L., & Jing, Y. (2021). Rank-sum ratio-based analysis on nursing human resource allocation in Sichuan Province in 2019. *Journal of Nursing*, 37(1), 68–72. (in China). 10.3760/cma.j.cn211501-20200528-02519

[cit0011] Jonassen, D. H. (2005). Tools for representing problems and the knowledge required to solve them. In S. O. Tergan & T. Keller (Eds.), *Knowledge and information visualization. Lecture notes in computer science*, Vol. 3426. Springer, 82–94 . 10.1007/11510154_5

[cit0012] Li, N. (2012). Current situation of nursing undergraduate education curriculum and practice teaching reform. *Journal of Bengbu Medical College*, 37(5), 610–612. 10.3969/j.1000-2200.2012.05.043

[cit0013] Li, Y. M., Huang, Y., Huang, Y., Sun, J. H., & Wei, D. D. (2020). Study on the relationship among resilience, problem solving and coping styles of clinical nurses. *Journal of Nursing Administration*, 20(5), 328–333. 10.3969/j.1671-315x.2020.05.006

[cit0014] Luo, Z. C., Bai, X. L., Zhong, Z. Y., & Li, J. Q. (2018). Qualitative study on attitude of PhD nursing students towards clinical work. *Chinese Journal of Hospital Administration*, 34(2), 157–161. 10.3760/cma.j.1000-6672.2018.02.016

[cit0015] Mayer, R. E., & Wittrock, M. C. (1992). *Thinking, problem solving, cognition* (2nd rev ed.). W H Freeman and Company.

[cit0016] McGuire, C. H. (1985). Medical problem-solving: A critique of the literature. *Journal of Medical Education*, 60(8), 587–595. https://kns.cnki.net/kcms/detail/detail.aspx?dbcode=CJFD&dbname=CJFDLAST2016&filename=YYGL201512035&uniplatform=NZKPT&v=tzIOR3BK5XPwrEGRv-zg3d5epVrkEKaR_t8heS4dRsIsdbx4lqFmp6xZBjJK0b-m 4020839

[cit0017] Mohammad, A. (2020). ”She’s dead!” - Nursing simulation practices: A discourse analysis approach. *Journal of Public Health Research*, 9(1), 1784. 10.4081/jphr.2020.1784 32582580 PMC7296278

[cit0018] National Development and Reform Commission. (2017). National nursing development plan (2016-2020). Retrieved July 20, 2017, from https://www.ndrc.gov.cn/fggz/fzzlgh/gjjzxgh/201707/t20170720_1196854.html?code=&state=123

[cit0019] Pan, W. (2016). Study on status quo of nurses’occupational attitude and its influencing factors. *Chinese Evidence-based Nursing*, 2(1), 14–16. 10.3969/j.issn.2095-8668.2016.01.04

[cit0020] Poghosyan, L., Ghaffari, A., Liu, J., & McHugh, M. D. (2020). Organizational support for nurse practitioners in primary care and workforce outcomes. *Nursing Research*, 69(4), 280–288. 10.1097/NNR.0000000000000425 32058457 PMC9869754

[cit0021] Skeriene, S. (2019). The integration of problem solving and value approach: The shift toward how to think. In L. Daniela (Ed.) *Innovations, Technologies and research in education*, (pp. 364–377). Association-for-Teacher-Education-in-Europe (ATEE) Spring conference.

[cit0022] Stone, M. (2020). Second victim support: Nurses’ perspectives of organizational support after an adverse event. *The Journal of Nursing Administration*, 50(10), 521–525. 10.1097/NNA.0000000000000928 32925663

[cit0023] Sun, Y. B., & Zong, M. B. (2017). Analysis of the training objectives of higher nursing professional education and the relevant problems. *Chinese Journal of Medical Education*, 37(6), 811–815. 10.3760/cma.j.1673-677X.2017.06.004

[cit0024] Taylor, C. (2000). Clinical problem-solving in nursing: Insights from the literature. *Journal of Advanced Nursing*, 31(4), 842–849. 10.1046/j.1365-2648.2000.01342.x 10759980

[cit0025] VanLehn, K. (1989). Problem-solving and cognitive skill acquisition. In M. L. Posner (Ed.), *Foundations of cognitive science*. The MIT Press, 1–47 .

[cit0026] Wang, X., Du, X., Liu, C., & Zhang, X. (2018). Does the professional attitude of physicians always affect their professional behaviour? A survey in tertiary hospitals in Nanchang City, China. *Australian Health Review: a Publication of the Australian Hospital Association*, 42(6), 650–655. 10.1071/AH16190 29566359

[cit0027] Wang, L. Q., Zhou, Y. J., Tang, L. Y. Q., Yan, W. J., Li, J. S., & Tang, X. M. (2021). Research on the construction of harmonious doctor-patient relationship under the environment of new media. *Military Medical Journal of South China*, 35(1), 54–56. 10.13730/j.issn.1009-2595.2021.01.014

[cit0028] Yang, H., Lu, Y. H., Qu, R. Y., Zhang, H., Zhou, J. J., & Bai, Y. L. (2019). The evaluation of practice and effective for evaluation configuration and nurse allocation based on nursing workload. *Journal of Nursing Administration*, 19(1), 57–60. https://kns.cnki.net/kcms/detail/detail.aspx?dbcode=CJFD&dbname=CJFDLAST2019&filename=HLGL201901015&uniplatform=NZKPT&v=R4prRQXZysjbCtP89P7wkR-_URdOoM7PoHJo6x6GwI9Zei9p2dRpmrperRmPnguL

[cit0029] Yang, Q. J., Yang, Q. J., & Huang, R. R. (2018). Status quo of postgraduate education model of master of nursing specialist. *Chinese Nursing Research*, 32(21), 3370–3372. 10.12102/j.1009-6493.2018.21.016

[cit0030] Zhang, G. F., Liu, Z. Y., Gao, S. J., Sun, N., & Feng, Y. P. (2019). The effects of scenario simulation on emergency response ability of junior nurses. *Chinese Journal of Nursing Education*, 16(7), 532–535. 10.3761/j.issn.1672-9234.2019.07.014

